# Supplementary material for: The Small RNA Universe of Capitella teleta
Source: Front Mol Biosci. 2022 Feb 25;9:802814. doi: 10.3389/fmolb.2022.802814 (PMC8915122; doi:10.3389/fmolb.2022.802814)
Supplement: Supplementary file 1 [file DataSheet1.ZIP › Supplement/known/CAPTEscaffold_114_8286.pdf]

Provisional ID : CAPTEscaffold_114_8286
Score total : 28150.9
Score for star read(s) : 3.9
Score for read counts : 28140.9
Score for mfe : 1.5
Score for randfold : 1.6
Score for cons. seed : 3
Total read count : 55209
Mature read count : 55144
Loop read count : 5
Star read count : 60

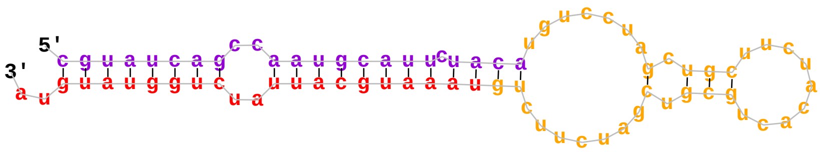


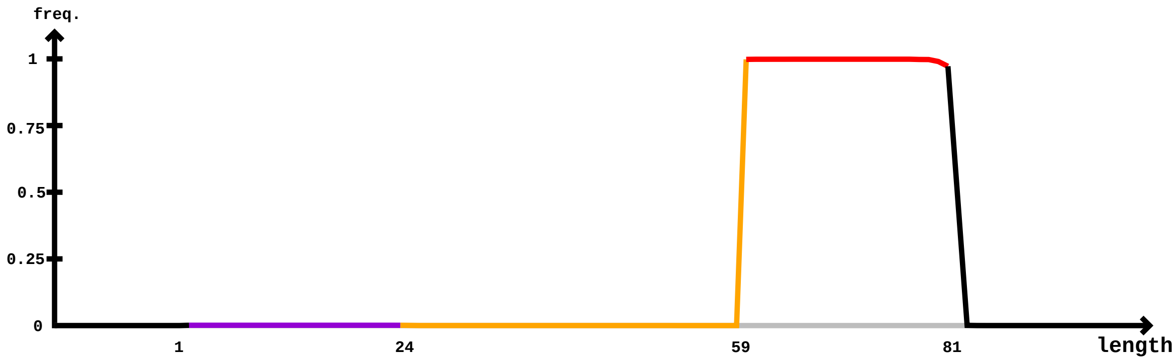


Star Mature

```
5'- cgucacuggugccguaucagccaaugcauucuacauguccuagcugcuucuacacugcgucgaucuucuguaaaugcauuaucugguauguaacucagugacgccucgccuc -3' obs
    cgucacuggugccguaucagccaaugcauucuacauguccuagcugcuucuacacugcgucgaucuucuguaaaugcauuaucugguauguaacucagugacgccucgccuc     exp
    cgucacuggugccguaucagccaaugcauucuacauguccuagcugcuucuacacugcgucgaucuucuguaaaugcauuaucugguauguaacucagugacgccucgccuc     known
    ((((((((((..((((((((..((((((((.((((.......(.(((.........))).).......))))))))))))..))))))))..)).))))))))......... reads mm sample
    ............cguaucagccaaugcauucu................................................................................ 3 0 seq
    ............cguaucagccaaugcauucuac.............................................................................. 2 0 seq
    ............cguaucagccaaugcauucuaca............................................................................. 54 0 seq
    ............cguaucGgccaaugcauucuaca............................................................................. 1 1 seq
    ............cguaucagccaaugcauucuacauguccu....................................................................... 1 0 seq
    ...........................auucuacauguccuagcugcuu............................................................... 1 0 seq
    ...................................uguccuagcugcuucuacacugcgucga................................................. 3 0 seq
    ...................................uguccuagcugcuucuacacugcgucgaucu.............................................. 2 0 seq
    .....................................................................Cuaaaugcauuaucugguaugua.................... 1 1 seq
    .....................................................................guaaaugcauuaucugguauguaacucagug............ 1 0 seq
    ......................................................................uaaaugcauuaucuggua........................ 56 0 seq
    ......................................................................uaaaugcauuGucugguau....................... 2 1 seq
    ......................................................................uaaaugcauuaucugguau....................... 14 0 seq
    ......................................................................uaaaugcauuGucugguaug...................... 6 1 seq
    ......................................................................uaaaugAauuaucugguaug...................... 1 1 seq
    ......................................................................uaaaugcauuaucugguauA...................... 4 1 seq
    ......................................................................uaaaugcauuaucugguaug...................... 385 0 seq
    ......................................................................uaaaugcaGuaucugguaug...................... 1 1 seq
    ......................................................................uaaaugcGuuaucugguaug...................... 2 1 seq
    ......................................................................uaaaugcauuaucugguaugu..................... 974 0 seq
    ......................................................................uaaaugcaAuaucugguaugu..................... 1 1 seq
    ......................................................................uaaaugcauuGucugguaugu..................... 6 1 seq
    ......................................................................uaaaugcauuaucugguaGgu..................... 1 1 seq
    ......................................................................Aaaaugcauuaucugguaugu..................... 3 1 seq
    ......................................................................Naaaugcauuaucugguaugu..................... 1 1 seq
    ......................................................................uaaaugcauCaucugguaugu..................... 1 1 seq
    ......................................................................uaaaugAauuaucugguaugu..................... 1 1 seq
    ......................................................................uaaaugcauuaucuAguaugu..................... 2 1 seq
    ......................................................................uaaaugcUuuaucugguaugu..................... 1 1 seq
    ......................................................................Gaaaugcauuaucugguaugu..................... 1 1 seq
    ......................................................................uaaaugcauuaAcugguaugu..................... 1 1 seq
    ......................................................................uaaauCcauuaucugguaugu..................... 1 1 seq
```

Star                                                   Mature

```
cgucacuggugccguaucagccaaugcauucuacauguccuagcugcuucuacacugcgucgaucuucuguaaaugcauuaucugguauguaacucagugacgccucgccuc
......................................................................uaaaugcauuaucAgguaugu.....................	1	1	seq
......................................................................uaaaugcauuaucugguaugA.....................	1	1	seq
......................................................................uaaaugcauAaucugguaugu.....................	1	1	seq
......................................................................uaaaugcauGaucugguaugua....................	2	1	seq
......................................................................uaaaugcUuuaucugguaugua....................	4	1	seq
......................................................................uaaaugcauuaucugguaugGa....................	6	1	seq
......................................................................uaaaugcauuauGugguaugua....................	2	1	seq
......................................................................uaaauAcauuaucugguaugua....................	7	1	seq
......................................................................uaaaugcaCuaucugguaugua....................	2	1	seq
......................................................................uaaaugcauuaucugguGugua....................	3	1	seq
......................................................................uaaaugcGuuaucugguaugua....................	14	1	seq
......................................................................uaaaugcauuaCcugguaugua....................	5	1	seq
......................................................................uaaGugcauuaucugguaugua....................	12	1	seq
......................................................................uaaaugcauuaucugguauCua....................	10	1	seq
......................................................................uaaaugcauuGucugguaugua....................	591	1	seq
......................................................................uaaauUcauuaucugguaugua....................	5	1	seq
......................................................................uaaaugcauuaAcugguaugua....................	19	1	seq
......................................................................uaaaGgcauuaucugguaugua....................	5	1	seq
......................................................................uaaaugcauuaucugUuaugua....................	2	1	seq
......................................................................Gaaaugcauuaucugguaugua....................	31	1	seq
......................................................................uaaaugcauuaucugguaAgua....................	13	1	seq
......................................................................uaaaugcauuauAugguaugua....................	23	1	seq
......................................................................uaaaCgcauuaucugguaugua....................	5	1	seq
......................................................................uaaaugcauuaucuggCaugua....................	4	1	seq
......................................................................uaaaugcauuaucGgguaugua....................	2	1	seq
......................................................................uaaaugcauuaucuggGaugua....................	5	1	seq
......................................................................uaaaugcauuaucugguauAua....................	5	1	seq
......................................................................uaaaugcauuaucugguaGgua....................	3	1	seq
......................................................................uaaaugcauuaucugguauguU....................	57	1	seq
......................................................................uaaaugcaAuaucugguaugua....................	14	1	seq
......................................................................uaaaugcauuaucugguCugua....................	1	1	seq
......................................................................uaaUugcauuaucugguaugua....................	5	1	seq
......................................................................uaaaAgcauuaucugguaugua....................	66	1	seq
......................................................................uaaaugcauuaucugguaugua....................	47664	0	seq
......................................................................uaaaugcauuaucugguaCgua....................	3	1	seq
......................................................................uaaaugcauuaucugguauguG....................	7	1	seq
......................................................................uaaaugcauuaucuCguaugua....................	7	1	seq
......................................................................Caaaugcauuaucugguaugua....................	7	1	seq
......................................................................Aaaaugcauuaucugguaugua....................	249	1	seq
......................................................................Naaaugcauuaucugguaugua....................	10	1	seq
......................................................................uaaaugUauuaucugguaugua....................	7	1	seq
......................................................................uaaaugcauuaucugguauUua....................	3	1	seq
......................................................................uaGaugcauuaucugguaugua....................	7	1	seq
......................................................................uaaauNcauuaucugguaugua....................	1	1	seq
......................................................................uaaaugcauuaucugguaugAa....................	20	1	seq
......................................................................uaaaugcauCaucugguaugua....................	6	1	seq
......................................................................uGaaugcauuaucugguaugua....................	16	1	seq
......................................................................uaaaugcauuaucugguUugua....................	4	1	seq
......................................................................uaaaugcaGuaucugguaugua....................	6	1	seq
......................................................................uaaauCcauuaucugguaugua....................	2	1	seq
......................................................................uaaaugcauuaucAgguaugua....................	14	1	seq
......................................................................uNaaugcauuaucugguaugua....................	2	1	seq
......................................................................uaaaugcauuaucuggAaugua....................	11	1	seq
......................................................................uaaaugcauuaucCgguaugua....................	7	1	seq
......................................................................uaUaugcauuaucugguaugua....................	4	1	seq
......................................................................uUaaugcauuaucugguaugua....................	1	1	seq
......................................................................uaaaugcauuaucugAuaugua....................	9	1	seq
......................................................................uaaaugcauuUucugguaugua....................	6	1	seq
......................................................................uaaaugcauuaucugguauguC....................	1	1	seq
......................................................................uaaaugcauAaucugguaugua....................	17	1	seq
......................................................................uaaaugcauuaucuAguaugua....................	93	1	seq
......................................................................uaaaugcauuaucuUguaugua....................	4	1	seq
......................................................................uaaaugAauuaucugguaugua....................	50	1	seq
......................................................................uaaaugGauuaucugguaugua....................	5	1	seq
......................................................................uaaaugcauuauUugguaugua....................	7	1	seq
......................................................................uaaaugcauuaucugguaugCa....................	34	1	seq
......................................................................uaaaugcauuaucugCuaugua....................	1	1	seq
......................................................................uaaaugcauuaucugguauguaG...................	3	1	seq
......................................................................uaaaugcauuaucugguauguaa...................	4285	0	seq
......................................................................uaaauUcauuaucugguauguaa...................	1	1	seq
```

Star Mature

```
cgucacuggugccguaucagccaaugcauucuacauguccuagcugcuucuacacugcgucgaucuucuguaaaugcauuaucugguauguaacucagugacgccucgccuc
......................................................................uaaaugcauuaucugguaugAaa................... 1 1 seq
......................................................................uaaaugcauuauAugguauguaa................... 3 1 seq
......................................................................uGaaugcauuaucugguauguaa................... 3 1 seq
......................................................................Aaaaugcauuaucugguauguaa................... 25 1 seq
......................................................................uaaaAgcauuaucugguauguaa................... 3 1 seq
......................................................................uaaaugAauuaucugguauguaa................... 4 1 seq
......................................................................uaaaugcauuGucugguauguaa................... 9 1 seq
......................................................................uaaaugcauuauUugguauguaa................... 1 1 seq
......................................................................uaaaugcauuaucuggAauguaa................... 2 1 seq
......................................................................uaaaugcauuaucuggCauguaa................... 1 1 seq
......................................................................uaaaugcauuaucAgguauguaa................... 1 1 seq
......................................................................uaaaugGauuaucugguauguaa................... 3 1 seq
......................................................................uaaaugcauuaCcugguauguaa................... 2 1 seq
......................................................................uaaaugcauuaucuAguauguaa................... 12 1 seq
......................................................................uaaaugcauuaucugguaCguaa................... 1 1 seq
......................................................................Gaaaugcauuaucugguauguaa................... 1 1 seq
......................................................................uaaaugcauAaucugguauguaa................... 4 1 seq
......................................................................uaaaugcGuuaucugguauguaa................... 1 1 seq
......................................................................uaaaugcauuaucGgguauguaa................... 1 1 seq
......................................................................uaaaugcaCuaucugguauguaa................... 1 1 seq
......................................................................uaaaugcauuaucugguauguGa................... 3 1 seq
......................................................................uaaaugcauuaAcugguauguaa................... 2 1 seq
......................................................................uaaaugcUuuaucugguauguaa................... 1 1 seq
......................................................................uaaaugcauuaucugCuauguaa................... 2 1 seq
......................................................................uaaaugcauuaucuCguauguaa................... 2 1 seq
......................................................................uaaaugcauuaucugguauguaU................... 13 1 seq
......................................................................uaaaugcauuaucugguaGguaa................... 1 1 seq
......................................................................uaaaugcauuaucugguaugCaa................... 1 1 seq
......................................................................uaaauAcauuaucugguauguaa................... 1 1 seq
......................................................................uaaaugcauCaucugguauguaa................... 2 1 seq
......................................................................uaaaugcauuaucugguauguaaA.................. 17 1 seq
......................................................................uaaaugcauuaucugguauguaaU.................. 13 1 seq
......................................................................uaaaugcauuaucugguauguaaUu................. 5 1 seq
.......................................................................Uaaugcauuaucugguaugua.................... 23 1 seq
.......................................................................aaaugcauuaucugguaugua.................... 1 0 seq
.......................................................................Uaaugcauuaucugguauguaa................... 2 1 seq
........................................................................Uaugcauuaucugguaugua.................... 1 1 seq
.........................................................................augcauuaucuAguaugua.................... 1 1 seq
.........................................................................augcauuaucugguaugua.................... 2 0 seq
.........................................................................augcauuaucugguauguaa................... 1 0 seq
..........................................................................ugcauuaucugguaugua.................... 2 0 seq
..........................................................................ugcauuaucugguauguaa................... 1 0 seq
...........................................................................gcauuaucugguauguaa................... 2 0 seq
.............................................................................................cucagugacgccucgccu. 1 0 seq
```
